# Author Correction: Single-cell RNA-seq reveals that glioblastoma recapitulates a normal neurodevelopmental hierarchy

**DOI:** 10.1038/s41467-020-17979-8

**Published:** 2020-08-07

**Authors:** Charles P. Couturier, Shamini Ayyadhury, Phuong U. Le, Javad Nadaf, Jean Monlong, Gabriele Riva, Redouane Allache, Salma Baig, Xiaohua Yan, Mathieu Bourgey, Changseok Lee, Yu Chang David Wang, V. Wee Yong, Marie-Christine Guiot, Hamed Najafabadi, Bratislav Misic, Jack Antel, Guillaume Bourque, Jiannis Ragoussis, Kevin Petrecca

**Affiliations:** 1 Department of Neurosciences, Montreal Neurological Institute-Hospital, McGill University, Montreal, QC Canada; 2 Department of Human Genetics, McGill University, Montreal, QC Canada; 3 McGill University and Genome Québec Innovation Centre, Montreal, QC Canada; 4 Canadian Centre for Computational Genomics, McGill University, Montreal, QC Canada; 5 Department of Clinical Neurosciences, University of Calgary, Calgary, AB Canada; 6 Department of Neuropathology, Montreal Neurological Institute and Hospital, McGill University, Montreal, QC Canada; 7 Department of Bioengineering, McGill University, Montreal, QC Canada

**Keywords:** Cancer stem cells, CNS cancer, Tumour heterogeneity

Correction to: *Nature Communications*10.1038/s41467-020-17186-5, published online 8 July 2020.

The original version of this Article contained an error in Fig. 1. On the x axis of Fig. 1e the labels for mesenchymal and astrocytic cells were swapped. This error has now been corrected in the Pdf and HTML versions of the Article.

