# Supplementary material for: Single-cell RNA-seq reveals that glioblastoma recapitulates a normal neurodevelopmental hierarchy
Source: Nat Commun. 2020 Jul 8;11:3406. doi: 10.1038/s41467-020-17186-5 (PMC7343844; doi:10.1038/s41467-020-17186-5)
Supplement: Supplementary file 1 — Supplementary information [file 41467_2020_17186_MOESM1_ESM.pdf]

Single-cell RNA-seq reveals that glioblastoma recapitulates a normal neurodevelopmental hierarchy

Couturier et al.

Supplementary Information

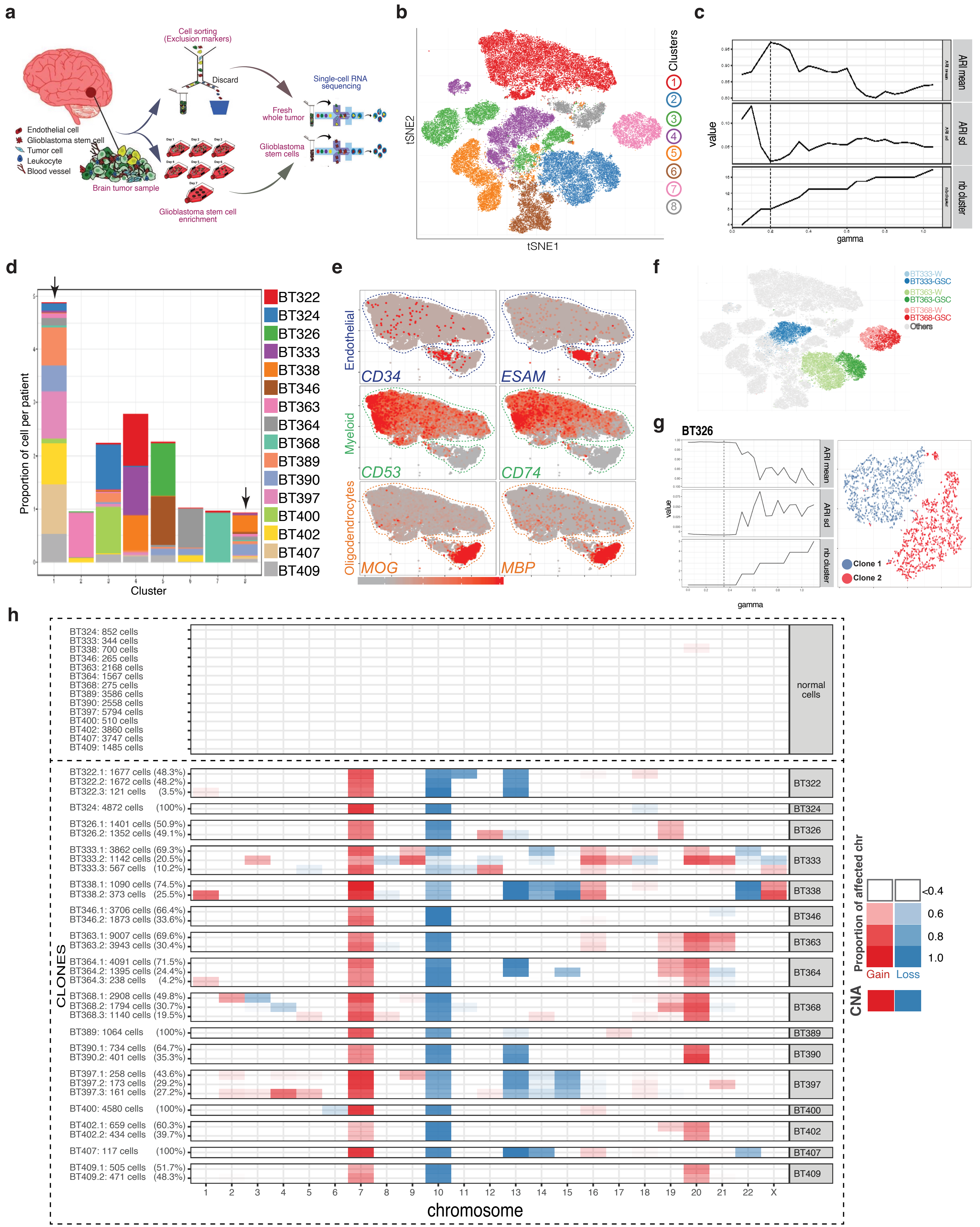
a
Cell sorting
(Exclusion markers)
Discard
Single-cell RNA
sequencing
Fresh
whole tumor
Glioblastoma
stem cells
Endothelial cell
Glioblastoma stem cell
Tumor cell
Leukocyte
Blood vessel
Brain tumor sample
Glioblastoma stem cell
enrichment
b
Clusters
1
2
3
4
5
6
7
8
tSNE2
tSNE1
c
ARI mean
ARI sd
nb cluster
value
gamma
d
Proportion of cell per patient
Cluster
BT322
BT324
BT326
BT333
BT338
BT346
BT363
BT364
BT368
BT389
BT390
BT397
BT400
BT402
BT407
BT409
e
Endothelial
CD34
ESAM
Myeloid
CD53
CD74
Oligodendrocytes
MOG
MBP
f
BT333-W
BT333-GSC
BT363-W
BT363-GSC
BT368-W
BT368-GSC
Others
g
BT326
ARI mean
ARI sd
nb cluster
value
gamma
Clone 1
Clone 2
h
BT324: 852 cells
BT333: 344 cells
BT338: 700 cells
BT346: 265 cells
BT363: 2168 cells
BT364: 1567 cells
BT368: 275 cells
BT389: 3586 cells
BT390: 2558 cells
BT397: 5794 cells
BT400: 510 cells
BT402: 3860 cells
BT407: 3747 cells
BT409: 1485 cells
normal
cells
BT322.1: 1677 cells (48.3%)
BT322.2: 1672 cells (48.2%)
BT322.3: 121 cells (3.5%)
BT322
BT324: 4872 cells (100%)
BT324
BT326.1: 1401 cells (50.9%)
BT326.2: 1352 cells (49.1%)
BT326
BT333.1: 3862 cells (69.3%)
BT333.2: 1142 cells (20.5%)
BT333.3: 567 cells (10.2%)
BT333
BT338.1: 1090 cells (74.5%)
BT338.2: 373 cells (25.5%)
BT338
BT346.1: 3706 cells (66.4%)
BT346.2: 1873 cells (33.6%)
BT346
BT363.1: 9007 cells (69.6%)
BT363.2: 3943 cells (30.4%)
BT363
BT364.1: 4091 cells (71.5%)
BT364.2: 1395 cells (24.4%)
BT364.3: 238 cells (4.2%)
BT364
BT368.1: 2908 cells (49.8%)
BT368.2: 1794 cells (30.7%)
BT368.3: 1140 cells (19.5%)
BT368
BT389: 1064 cells (100%)
BT389
BT390.1: 734 cells (64.7%)
BT390.2: 401 cells (35.3%)
BT390
BT397.1: 258 cells (43.6%)
BT397.2: 173 cells (29.2%)
BT397.3: 161 cells (27.2%)
BT397
BT400: 4580 cells (100%)
BT400
BT402.1: 659 cells (60.3%)
BT402.2: 434 cells (39.7%)
BT402
BT407: 117 cells (100%)
BT407
BT409.1: 505 cells (51.7%)
BT409.2: 471 cells (48.3%)
BT409
CLONES
1 2 3 4 5 6 7 8 9 10 11 12 13 14 15 16 17 18 19 20 21 22 X
chromosome
Proportion of affected chr
<0.4
0.6
0.8
1.0
Gain
Loss
CNA

Supplementary Figure 1 Experimental design and data processing.

(a) Workflow. Brain tumour samples were extracted and cells were freshly dissociated. These cells were either used for single-cell RNA-seq (whole tumour samples) or were cultured for 7 days to produce glioma stem cell enriched samples, which were then analyzed by single-cell RNA-seq. (b) t-distributed stochastic neighbour embedding (tSNE) of location-averaged transcriptome for all tumour cells (whole tumour and glioma stem cells) from 16 patients. Cells are colored by cluster. (c) Rand index by resolution parameter (gamma) for the clustering of location-averaged transcriptomic data. Top plot shows the mean Rand index, middle plot shows associated standard deviation, and bottom plot shows the median cluster number obtained for the associated gamma value. (d) Proportion of patient cells for each cluster seen in Supplementary Fig. 1b. Arrows point at the clusters containing normal cells (e) Expression of endothelial (CD34 and ESAM), myeloid (CD53 and CD74) and oligodendrocyte (MOG and MBP) genes in clusters devoid of CNAs (encircled clusters in Fig. 1a). (f) tSNE of location-averaged transcriptome for patients with paired GSC and whole tumour. (g) Example of determination of clonal population in a tumour. Rand index by resolution parameter (gamma) for the clustering of location-averaged transcriptomic data. Left: top plot shows the mean Rand index, middle plot shows associated standard deviation, and bottom plot shows the median cluster number obtained for the associated gamma value. Right: t-distributed stochastic neighbour embedding plot for this data with the cells colored by cluster/clone. (h) Copy number aberrations (CNA) heatmap for all main clones and normal cells of each patient. The transparency shows how much of the chromosome is affected, starting at 50%. All findings shown were significant ($p<0.001$) using the Wilcoxon test (two-sided).

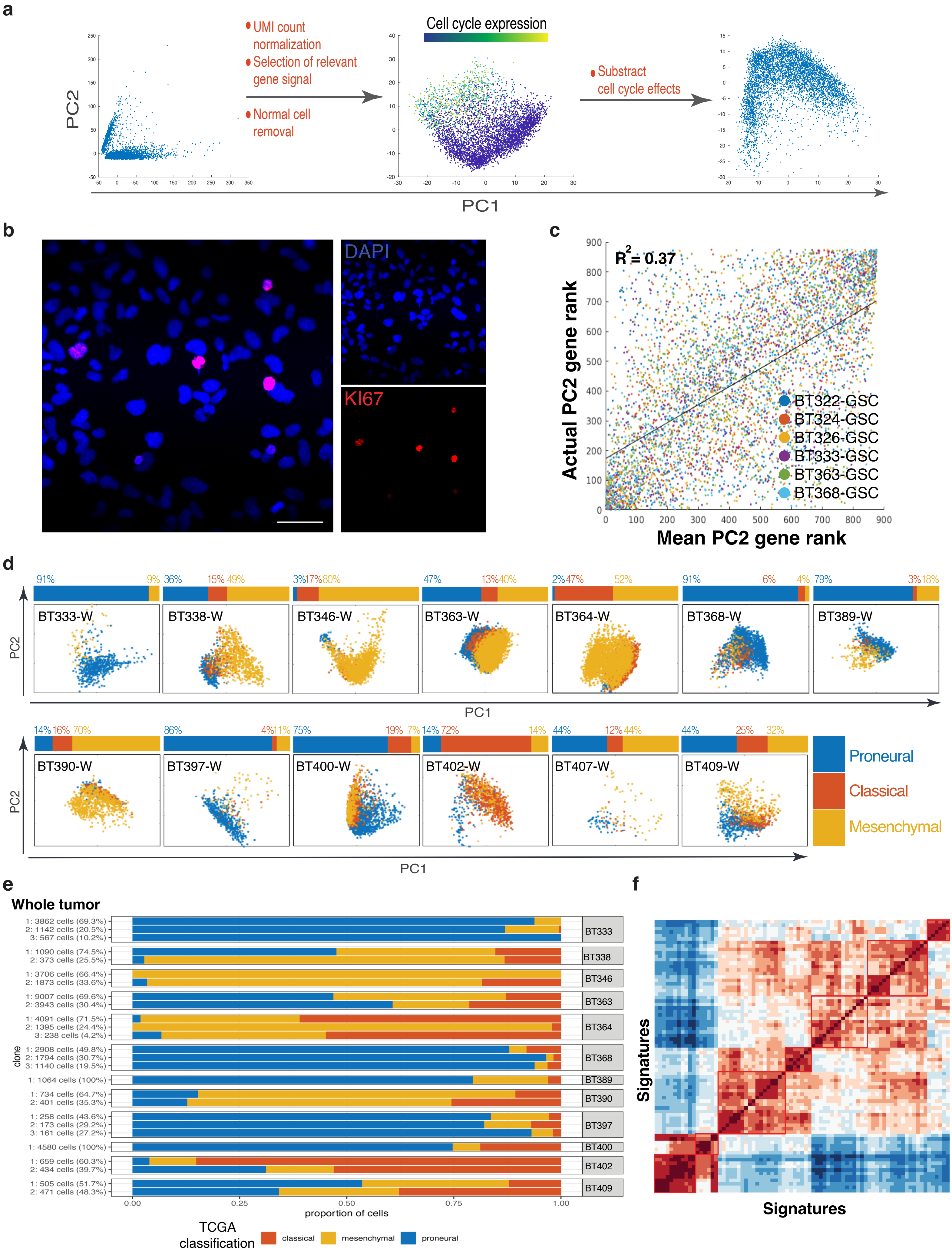

a
UMI count normalization
Selection of relevant gene signal
Normal cell removal
Cell cycle expression
Substract cell cycle effects
PC2
PC1
b
DAPI
KI67
c
R^2 = 0.37
Actual PC2 gene rank
Mean PC2 gene rank
BT322-GSC
BT324-GSC
BT326-GSC
BT333-GSC
BT363-GSC
BT368-GSC
d
BT333-W
BT338-W
BT346-W
BT363-W
BT364-W
BT368-W
BT389-W
BT390-W
BT397-W
BT400-W
BT402-W
BT407-W
BT409-W
Proneural
Classical
Mesenchymal
e
Whole tumor
1: 3862 cells (69.3%)
2: 1142 cells (20.5%)
3: 567 cells (10.2%)
BT333
1: 1090 cells (74.5%)
2: 373 cells (25.5%)
BT338
1: 3706 cells (66.4%)
2: 1873 cells (33.6%)
BT346
1: 9007 cells (69.6%)
2: 3943 cells (30.4%)
BT363
1: 4091 cells (71.5%)
2: 1395 cells (24.4%)
3: 238 cells (4.2%)
BT364
1: 2908 cells (49.8%)
2: 1794 cells (30.7%)
3: 1140 cells (19.5%)
BT368
1: 1064 cells (100%)
BT389
1: 734 cells (64.7%)
2: 401 cells (35.3%)
BT390
1: 258 cells (43.6%)
2: 173 cells (29.2%)
3: 161 cells (27.2%)
BT397
1: 4580 cells (100%)
BT400
1: 659 cells (60.3%)
2: 434 cells (39.7%)
BT402
1: 505 cells (51.7%)
2: 471 cells (48.3%)
BT409
clone
proportion of cells
TCGA classification
classical
mesenchymal
proneural
f
Signatures
Signatures

Supplementary Figure 2 Heterogeneity in glioblastoma.

(a) Bioinformatics workflow for normalization of gene expression data, gene selection, and removal of the cell cycle effect. Representative data for each step from BT324-GSC is shown by PCA. Raw data (left plot) is processed to remove low quality or normal cells and normalized for UMI count. Highly variable genes are selected. The resulting plot (middle) is characteristic for cell cycle effects as shown by the labelling of MKI67 expression in the cells. Removal of cell cycle effects yields the right plot. UMI: unique molecular identifier. (b) Representative immunofluorescence images showing Ki67 expression in enriched glioma stem cells after 1 week in culture. Image capture was done using a 20x objective with a further 2x digital magnification. n=6 biologically independent GSCs were sampled and stained. Scale bar: 50μm. (c) Expected and actual rank of genes by PC2 correlation. The actual gene rank (y-axis, one point per sample) correlates with the expected gene rank (x-axis) in all patients. (d) Cell-cycle corrected transcriptome principal component analysis of whole tumour cells for each patient. Cells are colored according to their TCGA subtype. Most TCGA subtypes were detected in each patient. (e) TCGA subtype by clone for each whole tumour sample. Differences in TCGA subtypes from clone to clone within a patient sample are statistically significant ($p<0.01$, Chi-Square test) in approximately 90% of the cases. (f) Heatmap showing the correlation between signatures obtained for all 13 whole-tumour samples. Signatures are ordered by according to the hierarchical clustering in Fig. 1e.

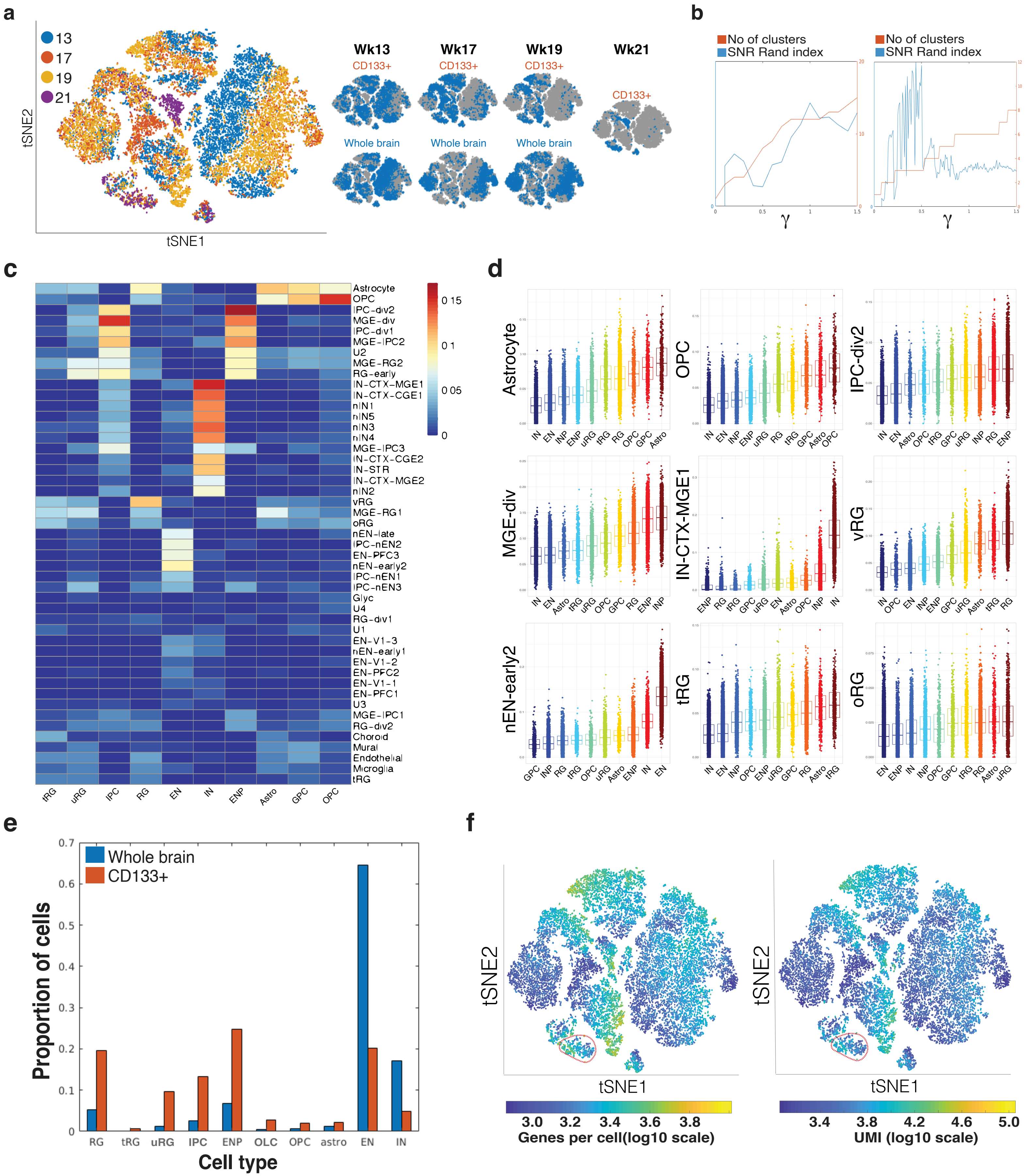
a
13
17
19
21
tSNE2
tSNE1
Wk13
Wk17
Wk19
Wk21
CD133+
Whole brain
b
No of clusters
SNR Rand index
γ
c
Astrocyte
OPC
IPC-div2
MGE-div
IPC-div1
MGE-IPC2
U2
MGE-RG2
RG-early
IN-CTX-MGE1
IN-CTX-CGE1
nIN1
nIN5
nIN3
nIN4
MGE-IPC3
IN-CTX-CGE2
IN-STR
IN-CTX-MGE2
nIN2
vRG
MGE-RG1
oRG
nEN-late
IPC-nEN2
EN-PFC3
nEN-early2
IPC-nEN1
IPC-nEN3
Glyc
U4
RG-div1
U1
EN-V1-3
nEN-early1
EN-V1-2
EN-PFC2
EN-V1-1
EN-PFC1
U3
MGE-IPC1
RG-div2
Choroid
Mural
Endothelial
Microglia
tRG
0.15
0.1
0.05
0
tRG
uRG
IPC
RG
EN
IN
ENP
Astro
GPC
OPC
d
Astrocyte
OPC
IPC-div2
MGE-div
IN-CTX-MGE1
vRG
nEN-early2
tRG
oRG
e
Whole brain
CD133+
Proportion of cells
Cell type
RG
tRG
uRG
IPC
ENP
OLC
OPC
astro
EN
IN
f
tSNE2
tSNE1
3.0
3.2
3.4
3.6
3.8
Genes per cell(log10 scale)
3.4
3.8
4.2
4.6
5.0
UMI (log10 scale)

Supplementary Figure 3 Clustering by cell type in whole and CD133-positive fetal brain cells.
(a) t-distributed stochastic neighbour embedding (t-SNE) map for the fetal dataset separated by gestational age (weeks) and CD133-sorting status. (b) Rand index signal-to-noise-ratio (SNR, blue) and number of clusters (red) as a function of resolution parameter (gamma, γ) for the full fetal dataset (left) and the glial population only (right). Peak SNRs value were chosen as a final clustering solution. (c) Heatmap showing similarity (Jaccard Coefficients) between reference clusters (rows), with clusters identified in the present study (columns). (d) Box plots showing the ranked-based similarity score (see Methods) between each cluster identified in the present study (Fig. 2a) and its corresponding top 9 (out of 47) reference clusters by similarity. The box plots were ordered from lowest to highest median score (highest similarity) and represent first quartile, median, and third quartile. (e) Proportion of cells by cell type for the whole brain and CD133-positive fetal samples. The GPC subgroup is enriched following CD133-sorting. (f) t-SNE of entire fetal dataset depicting the number of genes and UMI detected per cell. The circled cell group corresponds to GPCs.

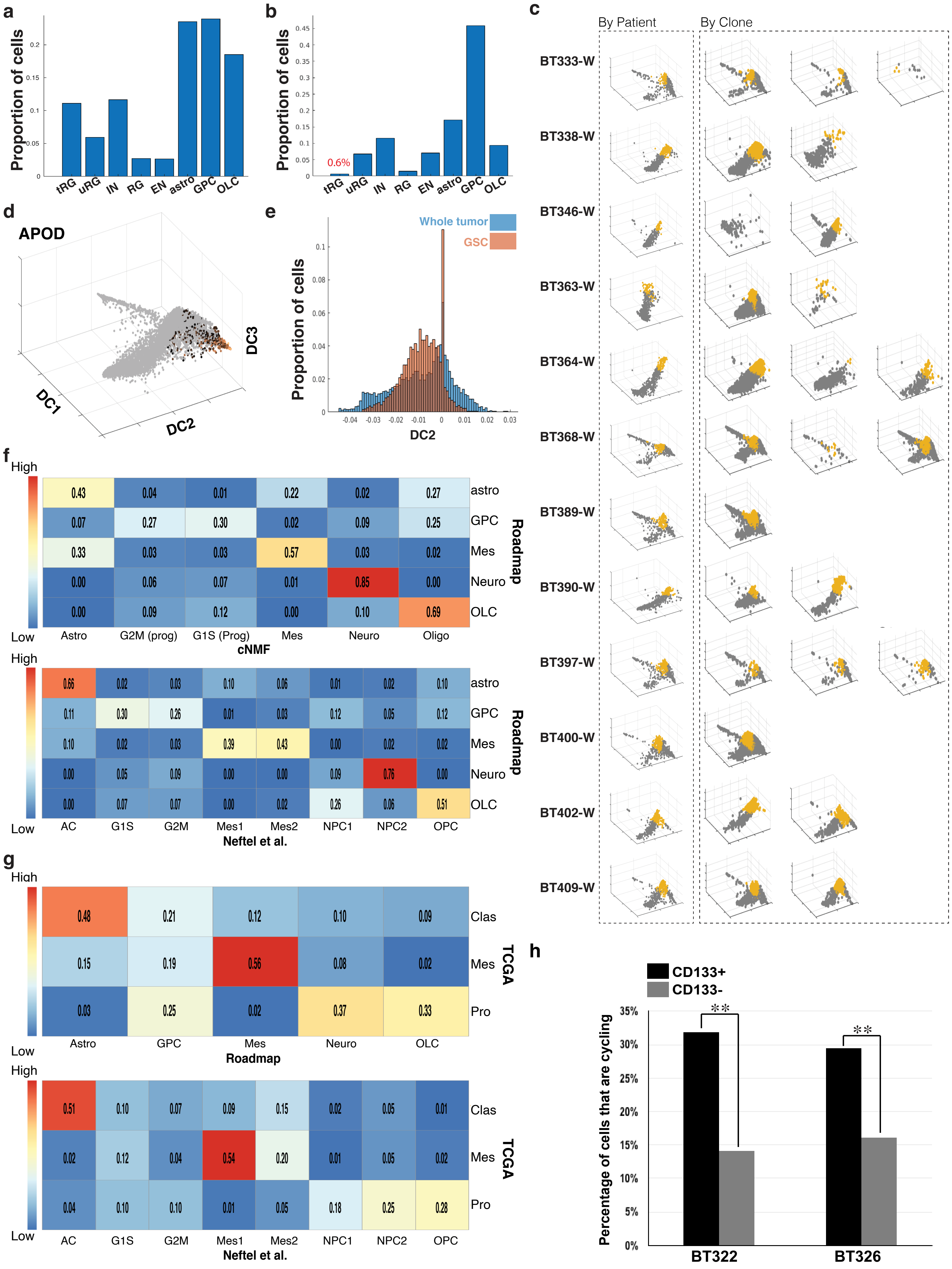

a
Proportion of cells
0.2
0.15
0.1
0.05
0
tRG
uRG
IN
RG
EN
astro
GPC
OLC
b
Proportion of cells
0.45
0.4
0.35
0.3
0.25
0.2
0.15
0.1
0.05
0
0.6%
tRG
uRG
IN
RG
EN
astro
GPC
OLC
c
By Patient
By Clone
BT333-W
BT338-W
BT346-W
BT363-W
BT364-W
BT368-W
BT389-W
BT390-W
BT397-W
BT400-W
BT402-W
BT409-W
d
APOD
DC1
DC2
DC3
e
Whole tumor
GSC
Proportion of cells
0.1
0.08
0.06
0.04
0.02
0
-0.04
-0.03
-0.02
-0.01
0
0.01
0.02
0.03
DC2
f
High
Low
0.43 0.04 0.01 0.22 0.02 0.27 astro
0.07 0.27 0.30 0.02 0.09 0.25 GPC
0.33 0.03 0.03 0.57 0.03 0.02 Mes
0.00 0.06 0.07 0.01 0.85 0.00 Neuro
0.00 0.09 0.12 0.00 0.10 0.69 OLC
Astro
G2M (prog)
G1S (Prog)
Mes
Neuro
Oligo
cNMF
Roadmap
High
Low
0.66 0.02 0.03 0.10 0.06 0.01 0.02 0.10 astro
0.11 0.30 0.26 0.01 0.03 0.12 0.05 0.12 GPC
0.10 0.02 0.03 0.39 0.43 0.00 0.02 0.02 Mes
0.00 0.05 0.09 0.00 0.00 0.09 0.76 0.00 Neuro
0.00 0.07 0.07 0.00 0.02 0.26 0.06 0.51 OLC
AC
G1S
G2M
Mes1
Mes2
NPC1
NPC2
OPC
Neftel et al.
Roadmap
g
High
Low
0.48 0.21 0.12 0.10 0.09 Clas
0.15 0.19 0.56 0.08 0.02 Mes
0.03 0.25 0.02 0.37 0.33 Pro
Astro
GPC
Mes
Neuro
OLC
Roadmap
TCGA
High
Low
0.51 0.10 0.07 0.09 0.15 0.02 0.05 0.01 Clas
0.02 0.12 0.04 0.54 0.20 0.01 0.05 0.02 Mes
0.04 0.10 0.10 0.01 0.05 0.18 0.25 0.28 Pro
AC
G1S
G2M
Mes1
Mes2
NPC1
NPC2
OPC
Neftel et al.
TCGA
h
CD133+
CD133-
Percentage of cells that are cycling
35%
30%
25%
20%
15%
10%
5%
0%
**
**
BT322
BT326

Supplementary Figure 4 Building a fetal roadmap to understand glioblastoma heterogeneity.
(a) Proportion of whole tumour cancer cells captured (see Results and Methods) by each fetal cell type. tRG: truncated radial glial; uRG: unknown radial glia; IN: interneurons (progenitors and more differentiated); RG: radial glia; EN: excitatory neurons (progenitors and more differentiated); astro: astrocytes; GPC: glial progenitor cells; OLC: oligo-lineage cells. (b) Proportion of glioma stem cells (GSCs) captured (see Results and Methods) by each fetal cell type. The tRG cell type captures a very low proportion of GSCs. See abbreviations in (a). (c) Whole tumour cancer cells isolated from 12 patients and mapped onto the roadmap in diffusion component space, by patient and by clone when applicable. Cells in yellow correspond to glial progenitor cancer cells. (d) Whole tumour cancer cells projected onto the roadmap showing expression of APOD. (e) Histogram of DC2 values for glioma stem cells (GSC) and whole tumour cells. A shift of cells from lower values (astrocytic) to intermediate values of DC2 occurs with GSC enrichment. Intermediate values of DC2 correspond to high values in DC3 and the glial progenitor cancer cell subtype. (f) Similarity analysis in our dataset of the fetal roadmap-based glioblastoma cell types, cNMF derived glioblastoma signatures (Fig. 1e), and the cell states describe by Neftel et al.44 (g) Comparison of the TCGA subtypes to the cell type signatures in the TCGA dataset. Cell type signatures were obtained using the roadmap and Neftel et al. TCGA subtypes were obtained using Gliovis. (h) Tumour immunolabeling, using Ki67 as a marker of cell proliferation, shows that the percentage of cycling cells in the CD133-positive population is significantly higher than that of CD133-negative population in two patients. Chi-square test: p values are 0.003 (GSC322, n=272 cells from 5 fields of view) and 0.006 (GSC326, n=319 cells from 5 fields of view). ** indicates $p<0.01$.

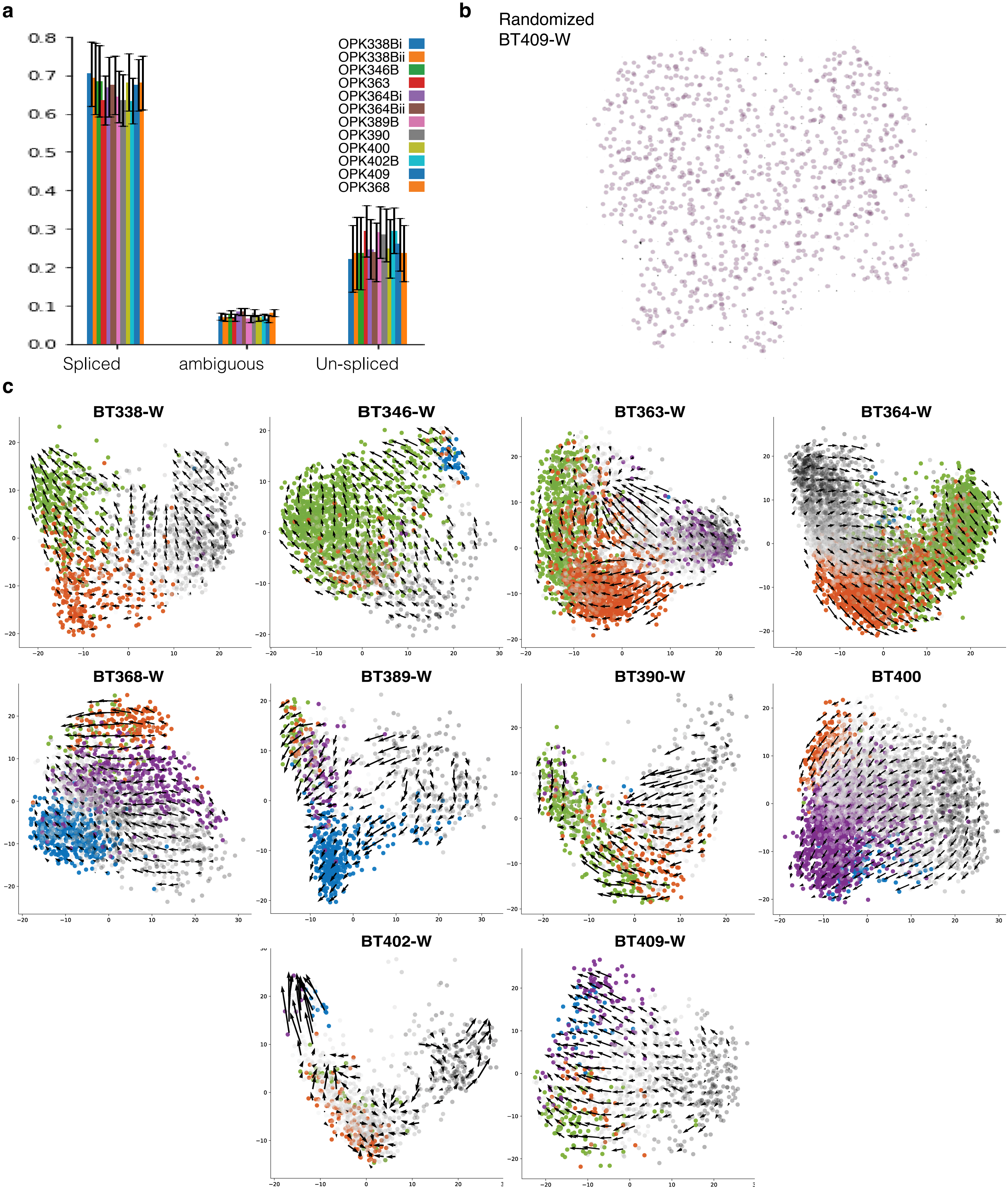

Supplementary Figure 5 RNA velocity of cancer cells.
(a) Proportions of spliced and unspliced RNA in each sample (n=5e5 to 5e7 per sample depending on number of cells and reads per sample) as measured by Velocyto plotted as mean +/- standard deviation. "i/ii" indicate samples that were captured twice. All samples contain 20 to 30% unspliced molecules (b) Representative sample of a velocity map with randomized data. No clear dynamic is discerned. (c) Velocity field superimposed to PC1 and PC2 embedding of cells for all samples. Cells are coloured by cell type according to Fig 5a.

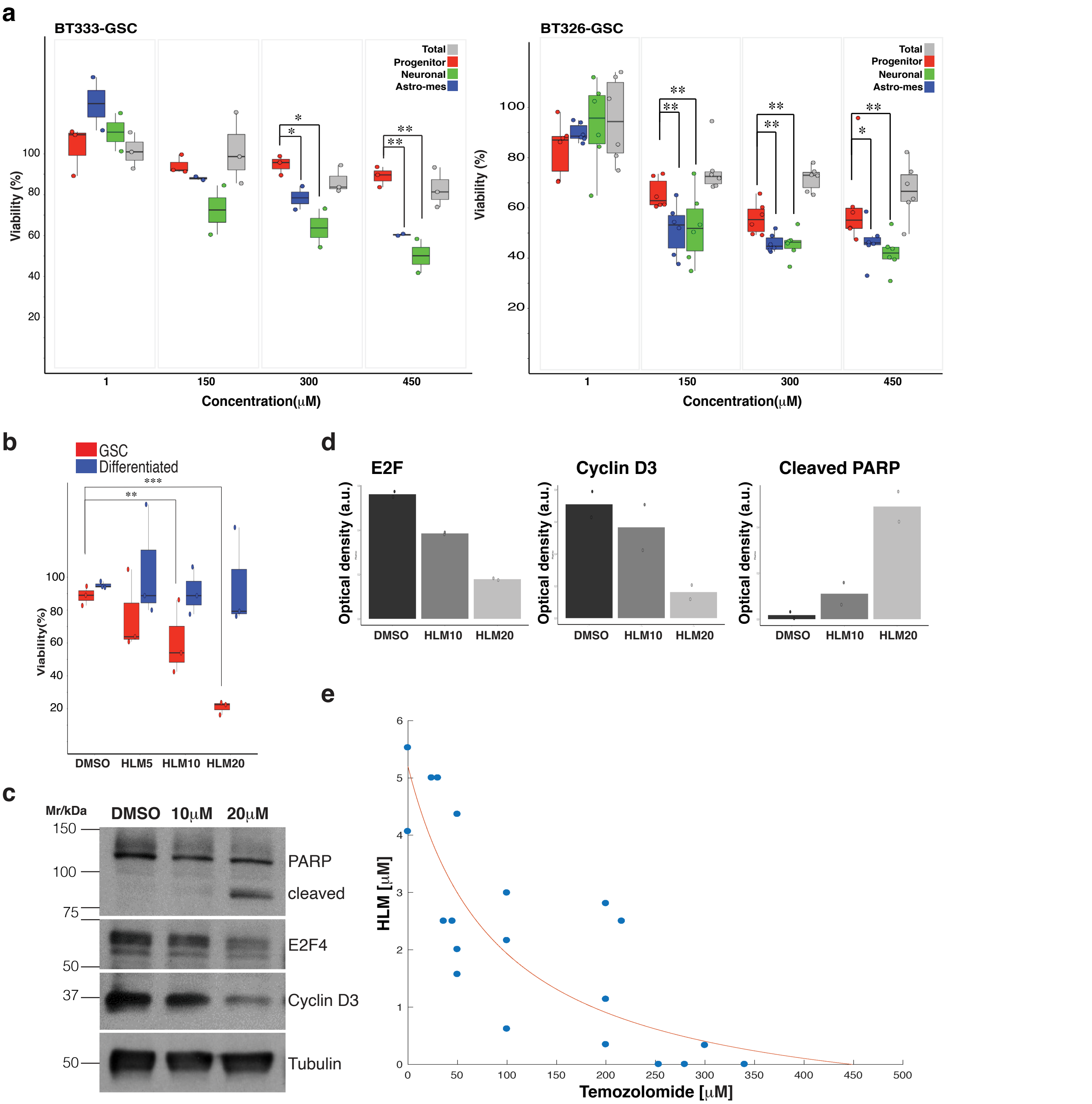

Supplementary Figure 6 Temozolomide assays show progenitor resistance and HLM006474 targets progenitors through inhibition of E2F4.

Supplementary Figure 6 Temozolomide assays show progenitor resistance and HLM006474 targets progenitors through inhibition of E2F4. (a) Box-whisker plots showing the proportion of viable glioma stem cells sorted by type followed by 5 days of temozolomide (TMZ) treatment, normalized to corresponding vehicle control. Each graph represents 1 patient sample with n = 2-3 biological replicates and 3 technical repeats for BT333-GSC and n=4-6 biological replicates and 3 technical replicates for BT326-GSC. Biological replicates are shown as overlaid dot plot. A one-tailed, two-sample equal variance t-test was used. *** indicates $p< 0.001$, ** indicates $p<0.01$, * indicates $p<0.05$. (b) A representative box-whisker plot showing the proportion of viable GSCs grown in serum free conditions or serum following 7 days of HLM006474 treatment, normalized to corresponding vehicle control. Data are represented as mean +/- SE; three experimental replicates (n=3) (p-values: DMSO-H10, 0.003; DMSO-H20, 4.4e-12). *** indicates $p<0.001$, ** indicates $p<0.01$ (one-tailed, two-sample t-test). n=5 independent biological replicates were done in total with technical triplicates and showed similar trend. (c) Levels of E2F4, cyclin D3, and cleaved PARP were analyzed by Western blot from two different GSC lines; representative data are shown. n=2 biologically independent GSC lines were analyzed. Full western blot found in Source Data. (d) Quantification of relative intensity of E2F4, cyclin D3, and cleaved PARP over tubulin are shown in the bar chart. n=2 biological replicates for each treatment group for each experiment. (e) Isobolographic analysis of HLM006474 and temozolomide. Data show the isobole for 40% efficiency in three biological repeats. Each data point is the average of three technical repeats. Curved line indicates the null, additive isobole at 40% efficiency. P-value = 0.74 (two-tailed t-test with n-1 degrees of freedom). All box plots represent the first quartile, median, and third quartile with whiskers corresponding to 1.5 times the interquartile range.

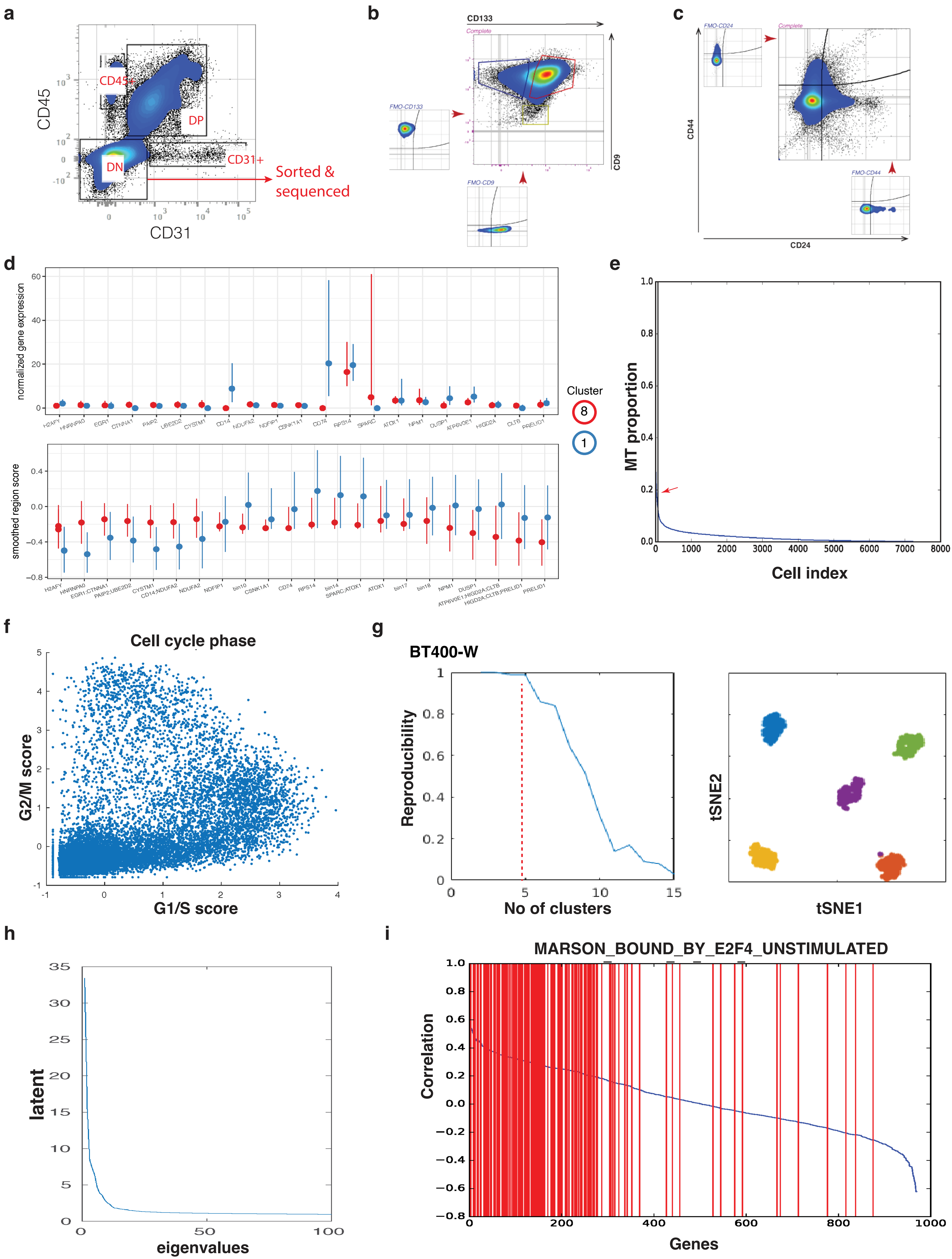

a
CD45
CD31
CD45+
DP
DN
CD31+
Sorted & sequenced
b
CD133
Complete
FMO-CD133
FMO-CD9
CD9
c
FMO-CD24
Complete
CD44
FMO-CD44
CD24
d
normalized gene expression
smoothed region score
Cluster
8
1
H2AFY
HNRNPA0
EGR1
CTNNA1
PAIP2
UBE2D2
CYSTM1
CD14
NDUFA2
NDFIP1
CSNK1A1
CD74
RPS14
SPARC
ATOX1
NPM1
DUSP1
ATP6V0E1
HIGD2A
CLTB
PRELID1
e
MT proportion
Cell index
f
Cell cycle phase
G2/M score
G1/S score
g
BT400-W
Reproducibility
No of clusters
tSNE2
tSNE1
h
latent
eigenvalues
i
MARSON_BOUND_BY_E2F4_UNSTIMULATED
Correlation
Genes

Supplementary Figure 7 Cytometry and bioinformatics processing.
(a) Gating strategy for primary patient samples dissociated with anti-CD45 and anti-CD31 antibodies to remove endothelial and immune cells. (b) and (c) show the gating strategy to sort glioma stem cells (Fig. 6, Fig. 7a-c and Fig. S6a). (c) gating strategy used in Fig. 1d. CD133 and CD9 double positive cells were also sorted for CD44 and CD24 double negative cells as depicted. FMO controls were used to set the gates. (d) Top plot - gene expression by cluster for genes present over a chromosomal region. Bottom plot - location-averaged gene expression is shown for the same region. An isolated but strong expression of CD74 in cluster 1 increases the location-averaged score in that region. For each gene, the point represents the median value across all samples (n=7 fetal samples) in the cluster while the vertical lines represent the interquartile range (25%-75% percentiles). (e) Proportion of unique molecular identifiers detected for mitochondrial genes. The vertical line (arrow) indicates the cut-off of 0.12. (f) Cell cycle plot showing G1/S and G2/M scores in the combined fetal dataset. Most cells have a value of 0 or less in both scores. (g) Example of the determination of number of signatures by cNMF (see Methods). Left: plot of reproducibility vs number of signatures. The reproducibility drops sharply below 0.9 beyond 5 signatures. Right: t-distributed stochastic neighbor embedding plot for all 100 repetitions of 5 signatures (500 signatures in total). Five well-defined clusters are apparent, highlighting the stability of the solution. (h) PCA eigenvalues for the combined fetal dataset. The first 10 eigenvalues have the highest values. (i) Example of an E2F gene set with strong enrichment in the progenitor cancer cell population. The blue line indicates the correlation of each gene for progenitor cancer cells vs astrocytic cancer cells. Red lines show the location of genes that are included in the gene set shown. The majority of genes present in the gene set correlate positively with progenitor as opposed to astrocytic cancer cells.
