# Supplementary material for: Single-cell RNA-seq reveals that glioblastoma recapitulates a normal neurodevelopmental hierarchy
Source: Nat Commun. 2020 Jul 8;11:3406. doi: 10.1038/s41467-020-17186-5 (PMC7343844; doi:10.1038/s41467-020-17186-5)
Supplement: Supplementary file 3 — Description of additional supplementary files [file 41467_2020_17186_MOESM3_ESM.pdf]

**Description of Additional Supplementary Files**

File Name: Supplementary Data 1
Description: Fetal brain differential gene expression by cell type.

File Name: Supplementary Data 2
Description: - Cancer gene expression in each diffusion component.

File Name: Supplementary Data 3
Description: Cancer gene order for the simplified PCA-based lineage and progenitor scores.

File Name: Supplementary Data 4
Description: - Gene set enrichment analysis of progenitor vs astro-mesenchymal cancer cells.
