# Supplementary figures and images for: Single-cell RNA-seq reveals that glioblastoma recapitulates a normal neurodevelopmental hierarchy
Source: Nat Commun. 2020 Jul 8;11:3406. doi: 10.1038/s41467-020-17186-5 (PMC7343844; doi:10.1038/s41467-020-17186-5)

Cyclin D3

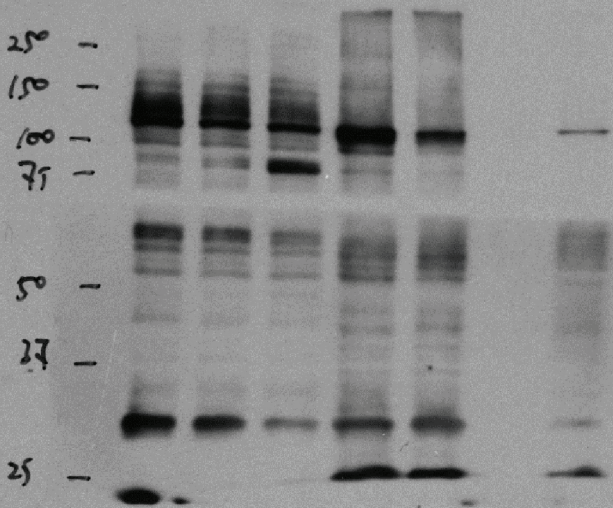

E2F4

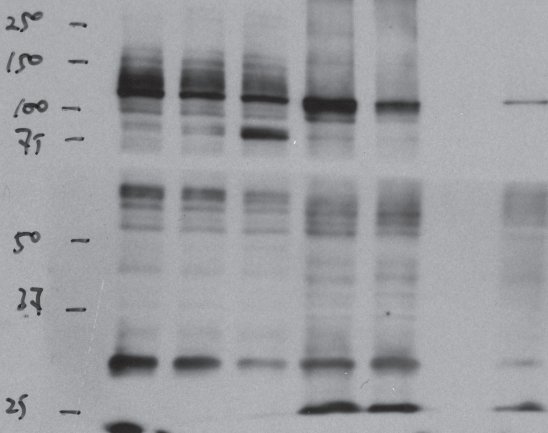

PARP/cleaved PARP

13/07/18

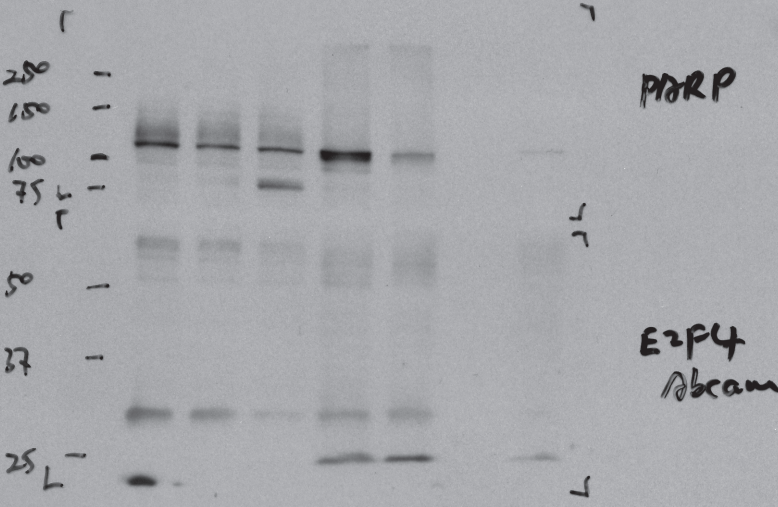

L DMSO 10 20 40 60 L HLM 20  
HLM 45 days  
after  
injection  
over night

Tubulin

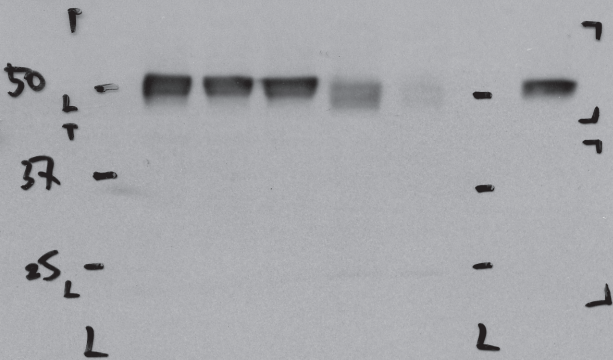

Supplement: Supplementary file 8 — Source data [file 41467_2020_17186_MOESM8_ESM.pdf]
