# Supplementary material for: Single-cell RNA-seq reveals that glioblastoma recapitulates a normal neurodevelopmental hierarchy
Source: Nat Commun. 2020 Jul 8;11:3406. doi: 10.1038/s41467-020-17186-5 (PMC7343844; doi:10.1038/s41467-020-17186-5)
Supplement: Supplementary file 9 — Reporting summary [file 41467_2020_17186_MOESM9_ESM.pdf]

nature research

Corresponding author(s): Dr. Kevin Petrecca

Last updated by author(s): May 8, 2020

Reporting Summary

Nature Research wishes to improve the reproducibility of the work that we publish. This form provides structure for consistency and transparency in reporting. For further information on Nature Research policies, see Authors & Referees and the Editorial Policy Checklist .

Statistics

For all statistical analyses, confirm that the following items are present in the figure legend, table legend, main text, or Methods section.

n/a	Confirmed	
☐	☒	The exact sample size (*n*) for each experimental group/condition, given as a discrete number and unit of measurement
☐	☒	A statement on whether measurements were taken from distinct samples or whether the same sample was measured repeatedly
☐	☒	The statistical test(s) used AND whether they are one- or two-sided<br>*Only common tests should be described solely by name; describe more complex techniques in the Methods section.*
☐	☒	A description of all covariates tested
☐	☒	A description of any assumptions or corrections, such as tests of normality and adjustment for multiple comparisons
☐	☒	A full description of the statistical parameters including central tendency (e.g. means) or other basic estimates (e.g. regression coefficient) AND variation (e.g. standard deviation) or associated estimates of uncertainty (e.g. confidence intervals)
☐	☒	For null hypothesis testing, the test statistic (e.g. *F*, *t*, *r*) with confidence intervals, effect sizes, degrees of freedom and *P* value noted<br>*Give P values as exact values whenever suitable.*
☒	☐	For Bayesian analysis, information on the choice of priors and Markov chain Monte Carlo settings
☒	☐	For hierarchical and complex designs, identification of the appropriate level for tests and full reporting of outcomes
☒	☐	Estimates of effect sizes (e.g. Cohen's *d*, Pearson's *r*), indicating how they were calculated

Our web collection on statistics for biologists contains articles on many of the points above.

Software and code

Policy information about availability of computer code

Data collection	No software was used for data collection.
Data analysis	scRNA-seq: cellRanger v2.2.0, custom scripts in Matlab (R2019a; packages: dpt, BCT v2017-15-1) and Python(v2.7 and 3.7; packages: scipy, matplotlib, numpy, pandas, velocyto); general statistics: R v3.4.4 (packages: splines, survival); Flow- and mass-cytometry: FlowJo v10

For manuscripts utilizing custom algorithms or software that are central to the research but not yet described in published literature, software must be made available to editors/reviewers. We strongly encourage code deposition in a community repository (e.g. GitHub). See the Nature Research guidelines for submitting code & software for further information.

Data

Policy information about availability of data

All manuscripts must include a data availability statement. This statement should provide the following information, where applicable:
- Accession codes, unique identifiers, or web links for publicly available datasets
- A list of figures that have associated raw data
- A description of any restrictions on data availability

EGA: EGAS00001004422; TCGA data: https://www.cancer.gov/about-nci/organization/ccg/research/structural-genomics/tcga; Neftel et al. GEO: GSE131928; Nowakoski et al.: bit.ly/cortexSingleCell

Field-specific reporting

Please select the one below that is the best fit for your research. If you are not sure, read the appropriate sections before making your selection.

☒ Life sciences ☐ Behavioural & social sciences ☐ Ecological, evolutionary & environmental sciences

For a reference copy of the document with all sections, see nature.com/documents/nr-reporting-summary-flat.pdf

Life sciences study design

All studies must disclose on these points even when the disclosure is negative.

Sample size	scRNA-seq : sixteen patients (GBM) and four patients (fetal) ; flow-cytometry : 8 patients (GBM) ; mass-cytometry : 1 patient (GBM); TMZ-assays : 3 patients (GBM) ; HLM-drug treatment : 16 mice ; Mouse survival by cancer cell type : 47
Data exclusions	No data was excluded.
Replication	All in vitro and in vivo findings were reproducible in three or more patient lines.
Randomization	Not applicable - study is not a clinical trial
Blinding	Not applicable - study is not a clinical trial

Reporting for specific materials, systems and methods

We require information from authors about some types of materials, experimental systems and methods used in many studies. Here, indicate whether each material, system or method listed is relevant to your study. If you are not sure if a list item applies to your research, read the appropriate section before selecting a response.

Materials & experimental systems

n/a	Involved in the study	
☐	☒	Antibodies
☒	☐	Eukaryotic cell lines
☒	☐	Palaeontology
☐	☒	Animals and other organisms
☐	☒	Human research participants
☐	☒	Clinical data

Methods

n/a	Involved in the study	
☒	☐	ChIP-seq
☐	☒	Flow cytometry
☒	☐	MRI-based neuroimaging

Antibodies

Antibodies used	CD133-PE(eBioscience, cat-12-1338-42), CD9-BV421 (BD, cat:743047), CD44-AF700(BD, cat: 561289), CD24-APC, CD45-BB515 (564)
Validation	Antibodies are commercially available and validated by using positive and negative control cells

Animals and other organisms

Policy information about studies involving animals; ARRIVE guidelines recommended for reporting animal research

Laboratory animals	Mice: NSG, NOD scid gamma, 14 days old at onset, female
Wild animals	Study did not involved wild animals
Field-collected samples	Study did not involved field-collected samples
Ethics oversight	The Neuro Animal Care Committee

Note that full information on the approval of the study protocol must also be provided in the manuscript.

Human research participants

Policy information about studies involving human research participants

Population characteristics	Male and female adult participants.
Recruitment	Patients were recruited consecutively in a single institution according to lab technical availability.
Ethics oversight	Montreal Neurological Hospital Research Ethics Board

Note that full information on the approval of the study protocol must also be provided in the manuscript.

Clinical data

Policy information about clinical studies

All manuscripts should comply with the ICMJEguidelines for publication of clinical research and a completedCONSORT checklist must be included with all submissions.

Clinical trial registration	*Provide the trial registration number from ClinicalTrials.gov or an equivalent agency.*
Study protocol	*Note where the full trial protocol can be accessed OR if not available, explain why.*
Data collection	*Describe the settings and locales of data collection, noting the time periods of recruitment and data collection.*
Outcomes	*Describe how you pre-defined primary and secondary outcome measures and how you assessed these measures.*

Flow Cytometry

Plots

Confirm that:

- [x] The axis labels state the marker and fluorochrome used (e.g. CD4-FITC).
- [ ] The axis scales are clearly visible. Include numbers along axes only for bottom left plot of group (a 'group' is an analysis of identical markers).
- [x] All plots are contour plots with outliers or pseudocolor plots.
- [ ] A numerical value for number of cells or percentage (with statistics) is provided.

Methodology

Sample preparation	The samples are human patient-derived. Each sample resected was treated with collagenase with magnesium chloride and DNase before being filtered through a 30um filter unit and subsequently passed through a density gradient medium to separate red blood cells and debris from cancer cells. The cells were then washed and counted and directly stained and sorted and the other half seeded in culture and subsequently stained and profiled via flow cytometry.
Instrument	BD LSR Fortessa - 5 lasers (355/405/488/561/640nm); 20 paramters analyzer<br>BD FACSAria III - 3 lasers (405/488/640 nm); 13 parameter cell sorter<br>BD FACSAria Fusion - 4 lasers (405/488/561/640 nm); 18 parameter cell sorter equipped with a biosafety cabinet
Software	Software used to acquire data on the instruments was FACS DIVA and FlowJo version 10 was used to analyze the data and plot the data.
Cell population abundance	During preliminary testing , sorted cells were stained with the same markers they were sorted with to determine post-sort purity.
Gating strategy	All samples were gated for cells using SSC/FSC and then gated for live cells using FSC/live-dead aqua. Finally singlets were acquired by plotting live cells using SSC-W/SSC-H or FSC-A/FSC-H.<br>For bulk sorting, singlets were subsequently gated for CD31 and CD45. Since only cells negative for these markers were collected post-sort, positive and negative population gating was done using only unstained control cells.<br>For multiparametric flow cytometry using patient-derived primary cultures, fluorescence-minus one controls were used to determine the cut-off points between negative and positive populations.

- [ ] Tick this box to confirm that a figure exemplifying the gating strategy is provided in the Supplementary Information.
